# The Case of the "Disappearing Ventricle": A Report

**DOI:** 10.7759/cureus.56525

**Published:** 2024-03-20

**Authors:** Turky Alsubahi, Sadiq Alqutub, Abdulsalam Alqutub

**Affiliations:** 1 Department of Anesthesiology, Faculty of Medicine, King Abdulaziz University, Jeddah, SAU; 2 Department of Pathology and Laboratory Medicine, Faculty of Medicine, King Abdulaziz University, Jeddah, SAU; 3 Department of Otolaryngology-Head and Neck Surgery, Faculty of Medicine, King Abdulaziz University, Jeddah, SAU

**Keywords:** transthoracic and transesophageal echocardiography, echocardiography, adult cardiac surgery, case report, vascular air embolism

## Abstract

Embolization of entrapped intracardiac air represents a significant risk to the patient undergoing open-heart surgery. Entrapment of as little as 0.5 mL of gas in the heart can cause temporary myocardial dysfunction, cardiac arrhythmias, and systemic emboli. In contrast, larger emboli can disrupt the evaluation of heart function by limiting visualization during echocardiography. We present the case of a 67-year-old male who presented with dizziness, nausea, and chest pain. A left heart catheterization revealed multi-vessel disease. Undergoing general anesthesia, the patient received three-vessel coronary artery bypass grafting, mitral valve repair, ring annuloplasty, and left atrial appendage closure. Upon aortic unclamping, transgastric echocardiography showed significant gas almost wholly obscuring the left heart chambers despite de-airing maneuvers. Successful resolution relied upon higher mean blood pressure and time, demonstrating the importance of intraoperative imaging and interdisciplinary collaboration.

## Introduction

Open-heart surgery poses the risk of air embolization, potentially leading to dysfunction or disability in vital organs [1]. The right upper pulmonary vein is the most common site of retained air or pooled air, followed by the left ventricle, left atrium, right coronary sinus of Valsalva, and left upper pulmonary vein [2]. Mitigation strategies, including carbon dioxide surgical field flooding and "de-airing" techniques, aim to reduce the likelihood of embolization [3]. Carbon dioxide is used for these maneuvers because it is about 16 times more soluble in blood than air. Open procedures involving heart structures, such as valves, ventricular outflow tracts, atrioventricular defects, tumors, and the aorta, may trap gases [4]. Intravenous injections or defects in interatrial/interventricular regions can introduce air into the heart. Even with careful "de-airing" procedures before separation from cardiopulmonary bypass, gas retained in the left heart chambers or aorta can embolize into coronaries as well as other vascular trees [5,6]. Entrapment of as little as 0.5 mL of gas in the heart can cause temporary myocardial dysfunction, cardiac arrhythmias, and systemic emboli. In contrast, larger emboli can disrupt the evaluation of heart function by limiting visualization during echocardiography [7]. This case report details an intracardiac air embolus almost entirely obscuring the left ventricular lumen.

## Case presentation

We present the case of a 67-year-old male with a history of coronary artery disease, hypertension, chronic kidney disease, and abdominal aortic aneurysm who presented with dizziness, nausea, and chest pain. After being ruled out for acute coronary events, a transesophageal echocardiogram (TEE) revealed a left ventricular ejection fraction (EF) of 65% and severe mitral regurgitation with an eccentric anteriorly directed jet. Left heart catheterization revealed multi-vessel disease, including 100% obstruction of the mid-distal right coronary artery and 80-90% obstruction of proximal and diagonal branches of the left anterior descending artery.

**Figure 1:**
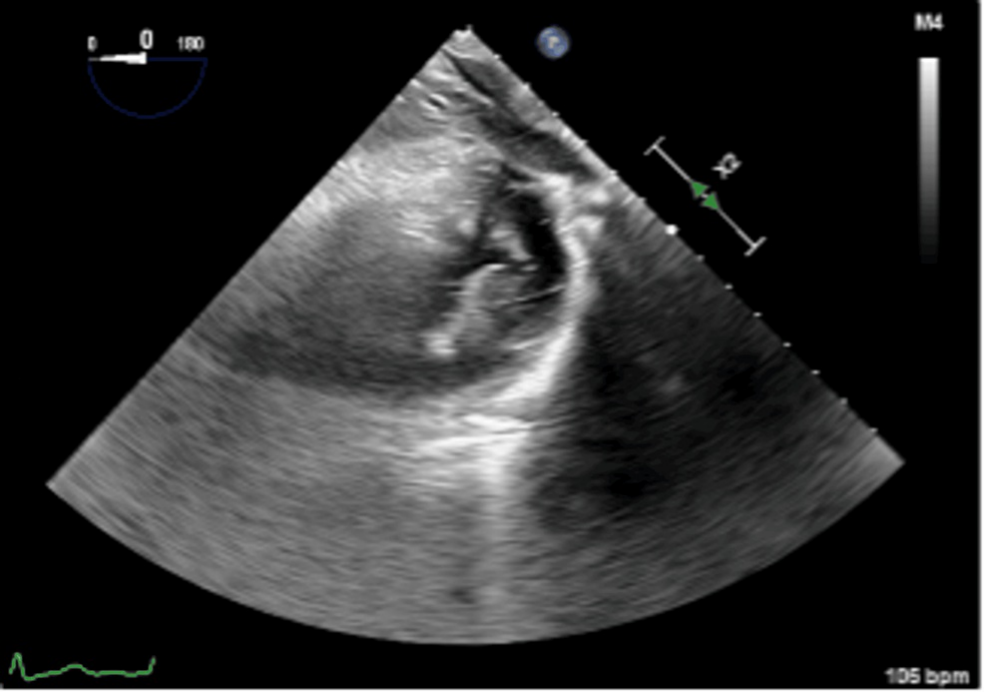
Transgastric short-axis view of the left ventricle. Note the cloudy left side of the image.

**Figure 2:**
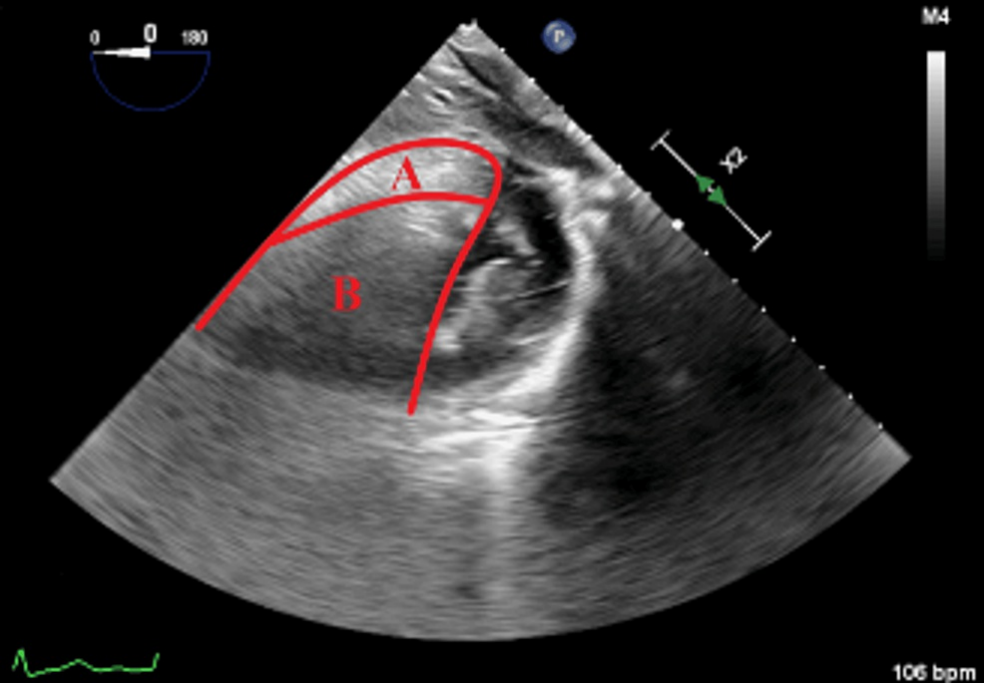
Transgastric short-axis view of the left ventricle. Note the cloudy left side of the image corresponding to the left ventricle inferior wall (A) and "cloud" (B) impeding the view of most of the left ventricular cavity as well as the septal and antero-septal walls.

**Figure 3:**
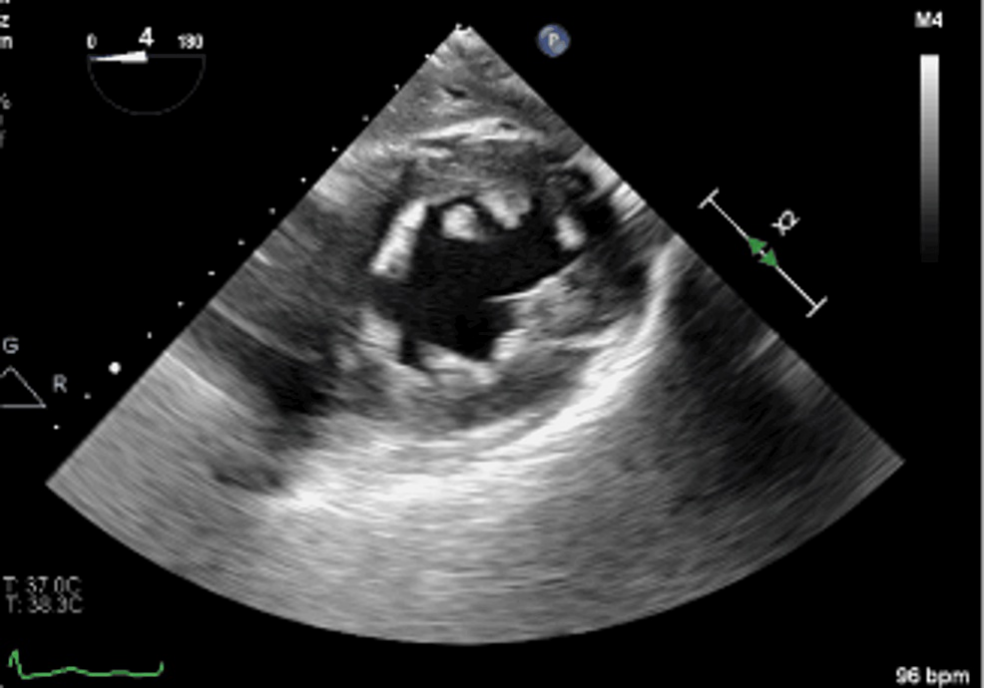
Transgastric short-axis view of the left ventricle. Note the disappearance of the cloudy area of the left ventricle once the gas has been "flushed" out of the coronary microvasculature within the myocardium.

Undergoing general anesthesia, the patient received three-vessel coronary artery bypass grafting, mitral valve repair with quadrangular resection, ring annuloplasty for a posterior middle segment flail leaflet with chordal rupture, and left atrial appendage closure. Upon aortic unclamping, significant gas was observed almost wholly obscuring the left heart chambers despite de-airing maneuvers. TEE had difficulty visualizing the left ventricle due to the cloud shadow appearance of the inferior wall of the left ventricle, with inferior wall dysfunction (estimated EF of 45%) (Figure 1 and Figure 2). Increasing mean arterial pressure and time led to gas disappearance from the myocardial vasculature, restoring normal function (EF of 65%) with no regional wall motion abnormalities (Figure 3). Weaning from cardiopulmonary bypass proceeded smoothly, and the postoperative course was uncomplicated.

This case illustrates common gas embolization into the right coronary artery (the most common coronary gas embolization due to its superior location in a supine patient) during open cardiac procedures. The resulting hazy image impedes visualization of the left ventricular inferior myocardial wall, partially or completely impeding visualization of distal territories. In our case, this was seen in the inferior, septal, and antero-septal regions. Successful resolution relied upon higher mean blood pressure and time, demonstrating the importance of intraoperative imaging and interdisciplinary collaboration.

## Discussion

The possibility and consequences of air entering the vascular system have long interested surgeons [8]. There are three potential ways that air in the left heart can cause death: (1) air entering the coronary arteries and obstructing them; (2) an air bolus in the ventricle itself that causes the cardiac pumping activity to fail; and (3) cerebral air embolism [9-12]. An essential concern to the patient undergoing open-heart surgery is the embolization of trapped intracardiac air after circulation is restored following cardiopulmonary bypass [13]. Trapped intracardiac air generates significant morbidity and mortality during cardiac surgery. These include cardiac chamber compression with varying cardiac placement, multiple chamber aspiration, and ventricular venting [14].

One potential method for identifying intracardiac air is echocardiography. The acoustic impedance of air and tissues differs significantly; therefore, at a tissue-air interface, an ultrasonic beam will be almost entirely reflected. The returning echocardiographic pattern should be considerably changed if an echocardiographic beam intercepts intracardiac air [1]. Due to the air's fast changes in location and appearance, TEE monitoring of retained intracardiac air, whether continuous or sequential, is very important in identifying the retained air, pinpointing its location, directing the air removal methods, and assessing the outcome through real-time monitoring [7]. Although echocardiography is also available, its use is intermittent and significantly disrupts the surgical procedure [1,15].

In cardiac procedures, the incidence of intracardiac air embolism is 73% in cardiac operations with cardiotomy and 11% in coronary artery bypass grafting [16]. Other procedures in other surgical fields were linked with intracardiac air. Although it is a rare complication, air embolism occurring during head and neck operations has been reported, significantly when the head is elevated during the procedure [17]. In another study on patients undergoing laparoscopic radical prostatectomy, by using TEE, gas embolisms were observed in 17.1% of the patients [4]. Gas embolism is associated with high rates of mortality, and the use of intraoperative imaging is recommended for detection and management [7,17,18]. 

## Conclusions

Open-heart surgery carries the potential risk of gas embolization, leading to significant morbidity and mortality. Even with careful "de-airing" procedures before separation from cardiopulmonary bypass, gas retained in the left heart chambers or aorta can embolize into coronaries. TEE is the imaging modality of choice for detection. Because intracardiac air rapidly changes its locations and appearance, continuous intraoperative TEE monitoring is essential, especially at weaning from bypass.

## References

[1] Duff HJ, Buda AJ, Kramer R, Strauss HD, David TE, Berman ND (1980). Detection of entrapped intracardiac air with intraoperative echocardiography. Am J Cardiol.

[2] Wellford AL, Lawrie G, Zoghbi WA (1996). Transesophageal echocardiographic features and management of retained intracardiac air in two patients after surgery. J Am Soc Echocardiogr.

[3] Padula RT, Eisenstat TE, Bronstein MH, Camishion RC (1971). Intracardiac air following cardiotomy: location, causative factors, and a method for removal. J Thorac Cardiovasc Surg.

[4] Hong JY, Kim WO, Kil HK (2010). Detection of subclinical CO2 embolism by transesophageal echocardiography during laparoscopic radical prostatectomy. Urology.

[5] Marco JD, Barner HB (1977). Aortic venting: comparison of vent effectiveness. J Thorac Cardiovasc Surg.

[6] Orihashi K, Ueda T (2019). "De-airing" in open heart surgery: report from the CVSAP nation-wide survey and literature review. Gen Thorac Cardiovasc Surg.

[7] Orihashi K, Matsuura Y, Hamanaka Y, Sueda T, Shikata H, Hayashi S, Nomimura T (1993). Retained intracardiac air in open heart operations examined by transesophageal echocardiography. Ann Thorac Surg.

[8] Geoghegan T, Lam CR (1953). The mechanism of death from intracardiac air and its reversibility. Ann Surg.

[9] Durant TM, Oppenheimer MJ, Webster MR, Long J (1949). Arterial air embolism. Am Heart J.

[10] Lewis FJ (1956). Hypothermia in surgery. J Natl Med Assoc.

[11] Moore RM, Braselton CW (1940). Injections of air and of carbon dioxide into a pulmonary vein. Ann Surg.

[12] Stegmann T, Daniel W, Bellmann L, Trenkler G, Oelert H, Borst HG (1980). Experimental coronary air embolism. Assessment of time course of myocardial ischemia and the protective effect of cardiopulmonary bypass. Thorac Cardiovasc Surg.

[13] Evans EA, Wellington JS (1964). Emboli associated with cardiopulmonary bypass. J Thorac Cardiovasc Surg.

[14] Ogawa S, Richardson JE, Sakai T, Ide M, Tanaka KA (2012). High mortality associated with intracardiac and intrapulmonary thromboses after cardiopulmonary bypass. J Anesth.

[15] Rodigas PC, Meyer FJ, Haasler GB, Dubroff JM, Spotnitz HM (1982). Intraoperative 2-dimensional echocardiography: ejection of microbubbles from the left ventricle after cardiac surgery. Am J Cardiol.

[16] Oka Y, Moriwaki KM, Hong Y, Chuculate C, Strom J, Andrews IC, Frater RW (1985). Detection of air emboli in the left heart by M-mode transesophageal echocardiography following cardiopulmonary bypass. Anesthesiology.

[17] Hybels RL (1980). Venous air embolism in head and neck surgery. Laryngoscope.

[18] Bonjer HJ, Hazebroek EJ, Kazemier G, Giuffrida MC, Meijer WS, Lange JF (1997). Open versus closed establishment of pneumoperitoneum in laparoscopic surgery. Br J Surg.

